# Construction of a reduced-order model based on tensor decomposition and its application to airbag deployment simulations

**DOI:** 10.1038/s41598-023-38393-2

**Published:** 2023-07-11

**Authors:** Takashi Sasagawa, Masato Tanaka

**Affiliations:** Toyota Central R&D Laboratories, Inc., 41-1 Yokomichi, Nagakute, Aichi 480-1192 Japan

**Keywords:** Mechanical engineering, Computational science

## Abstract

We present a construction method for reduced-order models (ROMs) to explore alternatives to numerical simulations. The proposed method can efficiently construct ROMs for non-linear problems with contact and impact behaviors by using tensor decomposition for factorizing multidimensional data and Akima-spline interpolation without tuning any parameters. First, we construct learning tensor data of nodal displacements or accelerations using finite element analysis with some representative parameter sets. Second, the data are decomposed into a set of mode matrices and one small core tensor using Tucker decomposition. Third, Akima-spline interpolation is applied to the mode matrices to predict values within the data range. Finally, the time history responses with new parameter sets are generated by multiplying the expanded mode matrices and small core tensor. The performance of the proposed method is studied by constructing ROMs for airbag impact simulations based on limited learning data. The proposed ROMs can accurately predict airbag deployment behavior even for new parameter sets using the Akima-spline interpolation scheme. Furthermore, an extremely high data compression ratio (more than 1000) and efficient predictions of the response surfaces and Pareto frontier (2000 times faster than that of full finite element analyses using all parameter sets) can be realized.

## Introduction

Owing to the increasing computational power in recent years, product design is typically realized by taking advantage of computer-aided engineering. Moreover, multi-scale analyses^1,2^ and multi-physics simulations^3^ for complex nonlinear or large-scale problems may also be performed in the product design process. However, the number of such expensive simulations is limited. Therefore, optimal design or uncertainty prediction based on multi-scale or multi-physics analyses cannot be realized due to the large number of computations required. For instance, simultaneously optimizing the structure design of multiple car models requires hundreds of hours, even though the K computer is used, where 10,000 or more large-scale crash simulations are required^4^.

The optimal solution of multiple design variables can be obtained through the response surface methodology, which derives the relationship between design variables and response values sampled in advance. However, constructing a response surface directly from the results of large-scale simulations, such as the relation between the material properties and deformation behavior of all elements obtained by high-fidelity simulations, is impossible. Hence, construction methods of reduced-order models (ROMs) based on proper orthogonal decomposition (POD) have been proposed in various fields, such as crash simulations^5^, aerodynamic optimization^6^, and parameterization of material microstructures^7^. Furthermore, ROMs based on POD are applied to structure-preserving algorithms of Hamiltonian systems^8^ and can provide high efficiency when combined with deep learning^9^. However, the POD is assimilated with principal component analysis or singular value decomposition, which is a low-rank factorization of two dimensional matrix data. Moreover, in most ROMs, POD projection coefficients are interpolated using the radial basis function (RBF), which requires tuning a shape parameter depending on the problem.

Tensor decomposition can factorize multidimensional data, which is a generalization of singular value decomposition. Tensor decomposition is known as the canonical polyadic (CP) model (also called PARAFAC or CandeComp)^10–12^, the Tucker model^13^, or the tensor-train decomposition^14^. In particular, a tensor describing multidimensional data is decomposed into a core tensor and a set of matrices in the Tucker decomposition. The core tensor is restricted to be superdiagonal in CP decomposition^15^; that is, the CP model is a special case of the Tucker model. Moreover, tensor decomposition has been applied to interpolate missing data scattered over a tensor^16–18^. Matrix multiplication for tensor decomposition can be efficiently computed with reinforcement learning^19^. An ROM based on hierarchical tensor approximation has been proposed for Neo-Hookean problems of a simple cube and plate with a hole^20^. Furthermore, spectral tensor-train decomposition^21^, which is based on functional tensor-train decomposition and polynomial approximations, has been applied to linear elliptic partial differential equations. However, its application to strongly non-linear problems (with contact and impact behaviors) and multi-objective optimization problems has not been demonstrated yet to the best of the authors’ knowledge. Furthermore, investigations on suitable interpolation methods for the ROMs of strongly non-linear problems are limited.

This study aims to rapidly predict the response surfaces and Pareto frontier to reduce product development cost and time. To achieve this purpose, a construction method of the ROM for multidimensional data is proposed using tensor decomposition instead of POD. Moreover, the response surfaces and Pareto frontier in multiple design spaces are efficiently predicted without tuning parameters using piecewise linear or Akima-spline interpolation^22^ instead of the RBF. This paper is organized as follows. Firstly, we present the formulation of the CP and Tucker models and then propose the construction method of the ROM using piecewise linear or Akima-spline interpolation. Secondly, we provide a numerical example of the prediction of the response surfaces and Pareto frontier in multiple design spaces with the proposed ROM. Finally, conclusions are summarized.

## Construction method of reduced-order model

As previously mentioned, CP and Tucker decomposition are typical in tensor decomposition. First, the CP and Tucker models are formulated, and the construction method of the ROM is then proposed using tensor decomposition with some interpolation methods.

### Tensor decomposition

The formulation of the tensor decomposition for three-dimensional data ${\bar{{\varvec{\mathcal {X}}}}}^{\text{3D}} \in {{\mathbb {R}}}^{I \times J \times K}$ is shown as follows. The CP model for ${\bar{{\varvec{\mathcal {X}}}}}^{\text{3D}}$ is defined as

1 $$\begin{aligned} {\bar{{\mathcal {X}}}_{ijk}}^{\text{3D}} = {{\mathcal {X}}}_{ijk}^{{\text{CP}}} + \mathcal{E}_{ijk}^{{\text{CP}}} = \sum \limits _{r = 1}^R {A_{ir}^{{\text{CP}}}B_{jr}^{{\text{CP}}}C_{kr}^{{\text{CP}}}} + \mathcal{E}_{ijk}^{{\text{CP}}}, \end{aligned}$$

where ${{\varvec{{\mathcal {X}}}}}^{\mathrm{{CP}}} \in {{\mathbb {R}}}^{I \times J \times K}$ denotes the low-rank three-dimensional data obtained by the CP model, and ${{\varvec{A}}}^{\mathrm{{CP}}} \in {{\mathbb {R}}}^{I \times R}, {{\varvec{B}}}^{\mathrm{{CP}}} \in {{\mathbb {R}}}^{J \times R}, {{\varvec{C}}}^{\mathrm{{CP}}} \in {{\mathbb {R}}}^{K \times R}$ are the mode matrices corresponding to each dimension *i*, *j*, *k* of ${\bar{{\varvec{\mathcal {X}}}}}^{\text{3D}}$, respectively. In addition, ${{\varvec{{\mathcal {E}}}}}^{\mathrm{{CP}}} \in {{\mathbb {R}}}^{I \times J \times K}$ is the decomposition error caused by reducing the rank of ${\bar{{\varvec{\mathcal {X}}}}}^{\text{3D}}$ using the CP model. In this study, ${\bar{{\varvec{\mathcal {X}}}}}$ is the learning data, implying full-rank data, and ${{\varvec{{\mathcal {X}}}}}$ indicates low-rank data.

The Tucker model for ${\bar{{\varvec{\mathcal {X}}}}}^{\text{3D}}$ is defined as

2 $$\begin{aligned} {\bar{{\mathcal {X}}}_{ijk}}^{\text{3D}} = {{\mathcal {X}}}_{ijk}^{{\text{Tucker}}} + {{\mathcal {E}}}_{ijk}^{{\text{Tucker}}} = \sum \limits _{s = 1}^S {\sum \limits _{u = 1}^U {\sum \limits _{v = 1}^V {A_{is}^{{\text{Tucker}}}B_{ju}^{{\text{Tucker}}}C_{kv}^{{\text{Tucker}}}{G_{suv}}} } } + {{\mathcal {E}}}_{ijk}^{{\text{Tucker}}}, \end{aligned}$$

where ${{\varvec{{\mathcal {X}}}}}^{\mathrm{{Tucker}}} \in {{\mathbb {R}}}^{I \times J \times K}$ is the low-rank three-dimensional data obtained by the Tucker model, ${{\varvec{A}}}^{\mathrm{{Tucker}}} \in {{\mathbb {R}}}^{I \times S}, {{\varvec{B}}}^{\mathrm{{Tucker}}} \in {{\mathbb {R}}}^{J \times U}, {{\varvec{C}}}^{\mathrm{{Tucker}}} \in {{\mathbb {R}}}^{K \times V}$ are the mode matrices corresponding to each dimension *i*, *j*, *k* of ${\bar{{\varvec{\mathcal {X}}}}}^{\text{3D}}$, respectively, and ${{\varvec{{\mathcal {E}}}}}^{\mathrm{{CP}}} \in {{\mathbb {R}}}^{I \times J \times K}$ is the decomposition error produced by reducing the rank of ${\bar{{\varvec{\mathcal {X}}}}}^\text{3D}$ using the Tucker model. Furthermore, the core tensor ${{\varvec{G}}} \in {{\mathbb {R}}}^{S \times U \times V}$ describes the interaction among the mode matrices ${{\varvec{A}}}^{\mathrm{{Tucker}}}, {{\varvec{B}}}^{\mathrm{{Tucker}}}, {{\varvec{C}}}^{\mathrm{{Tucker}}}$. Equation (2) is equal to Eq. (1) when $S = U = V$, and ${{\varvec{G}}}$ is equal to the superdiagonal tensor ${{\varvec{T}}} \in {{\mathbb {R}}}^{S \times U \times V}$ defined as

3 $$\begin{aligned} {T_{suv}} = \left\{ \begin{array}{l} \,1,\;\;\;\;\;{\text{if}}\;\;\;s = u = v,\\ \,0,\;\;\;\;\;{\text{otherwise}}. \end{array} \right. \end{aligned}$$

Therefore, the mode matrices of the CP model are independent of each other, and the interaction among the mode matrices is considered in the Tucker model.

The above formulation can be easily generalized to tensor decomposition for arbitrary *n*-dimensional data. In one example, the formulation of the tensor decomposition for four-dimensional data ${\bar{{\varvec{\mathcal {X}}}}}^{\text{4D}} \in {{\mathbb {R}}}^{I \times J \times K \times L}$ is shown.

4 $$\begin{aligned} {\bar{{\mathcal {X}}}_{ijkl}}^{\text{4D}}= & {} {{\mathcal {X}}}_{ijkl}^{{\text{CP}}} + {{\mathcal {E}}}_{ijkl}^{{\text{CP}}} = \sum \limits _{r = 1}^R {A_{ir}^{{\text{CP}}}B_{jr}^{{\text{CP}}}C_{kr}^{{\text{CP}}}D_{lr}^{{\text{CP}}}} + {{\mathcal {E}}}_{ijkl}^{{\text{CP}}} \end{aligned}$$

5 $$\begin{aligned} {\bar{{\mathcal {X}}}_{ijkl}}^{\text{4D}}= & {} \mathcal{X}_{ijkl}^{{\text{Tucker}}} + {{\mathcal {E}}}_{ijkl}^{{\text{Tucker}}} = \sum \limits _{s = 1}^S {\sum \limits _{u = 1}^U {\sum \limits _{v = 1}^V {\sum \limits _{w = 1}^W {A_{is}^{{\text{Tucker}}}B_{ju}^{{\text{Tucker}}}C_{kv}^{{\text{Tucker}}}D_{lw}^{{\text{Tucker}}}{G_{suvw}}} } } } + {{\mathcal {E}}}_{ijkl}^{{\text{Tucker}}} \end{aligned}$$

The target of the tensor decomposition explained below returns to three-dimensional data.

The mode matrices and core tensor of the CP and Tucker models are obtained by solving the following optimization problems:

6 $$\begin{aligned}{} & {} \mathop {\min }\limits _{{{\varvec{A}}},{{\varvec{B}}},{{\varvec{C}}}} \left\| {{{\varvec{\bar{{\mathcal {X}}}}}}^{\text{3D}} - {{\varvec{{\mathcal {X}}}}}^{\mathrm{{CP}}}\left( {{{{\varvec{A}}}^{{\text{CP}}}},{{{\varvec{B}}}^{{\text{CP}}}},{{{\varvec{C}}}^{{\text{CP}}}}} \right) } \right\| , \end{aligned}$$

7 $$\begin{aligned}{} & {} \mathop {\min }\limits _{{{\varvec{A}}},{{\varvec{B}}},{{\varvec{C}}},{{\varvec{G}}}} \left\| {{\bar{{\varvec{\mathcal {X}}}}}^{\text{3D}} - {{\varvec{{\mathcal {X}}}}}^{\mathrm{{Tucker}}}\left( {{{{\varvec{A}}}^{{\text{Tucker}}}},{{{\varvec{B}}}^{{\text{Tucker}}}},{{{\varvec{C}}}^{{\text{Tucker}}}},{{\varvec{G}}}} \right) } \right\| , \end{aligned}$$

where $\left\| \cdot \right\|$ corresponds to the Frobenius norm. Although these optimization problems are solved using alternating least squares^11,13^, several local minima exist. Therefore, the initial values in the optimization are computed using higher-order singular value decomposition^23,24^.

The core consistency is defined as

8 $$\begin{aligned} {\text{core}}\;{\text{consistency}} = 100 \times \left( {1 - \frac{{\sum \nolimits _{s = 1}^S {\sum \nolimits _{u = 1}^U {\sum \nolimits _{v = 1}^V {{{\left( {{T_{suv}} - {G_{suv}}} \right) }^2}} } } }}{{\sum \nolimits _{s = 1}^S {\sum \nolimits _{u = 1}^U {\sum \nolimits _{v = 1}^V {G_{suv}^2} } } }}} \right) . \end{aligned}$$

CP decomposition is appropriate when the core consistency is close to 100% because the core tensor is almost superdiagonal. However, the Tucker model is suitable when the core consistency is negative or close to 0%^15^.

### Prediction based on tensor decomposition

**Figure 1:**
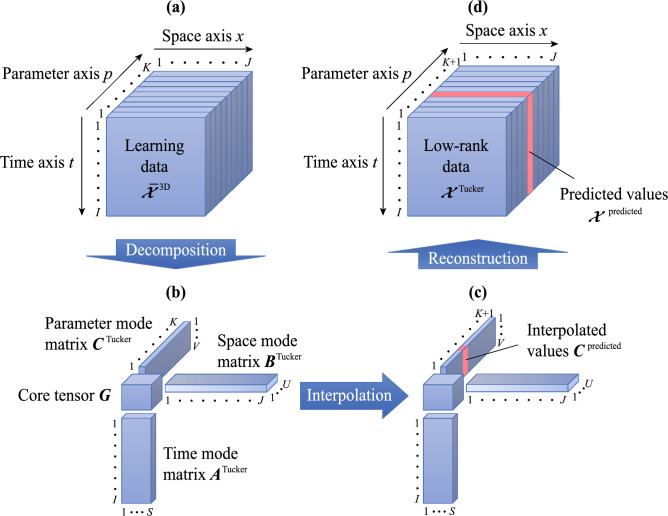
Conceptual diagram of the proposed method to predict multidimensional data based on tensor decomposition.

Here, two methods using piecewise linear and Akima-spline interpolation are proposed to predict multidimensional data based on the mode matrices and core tensor obtained by tensor decomposition. Although the cubic-spline interpolation is normally used, the Akima-spline interpolation is applied in our method to avoid overshooting when using cubic-spline interpolation. In addition, because the CP model is a special case of the Tucker model, only prediction methods using the Tucker model are presented below. Our method can be applied to ($N + 2$)-dimensional data comprising a time axis, space axis, and *N* parameter axes. The conceptual diagram of the proposed method when $N = 1$ is depicted in Fig. 1. First, ${\bar{{\varvec{\mathcal {X}}}}}^{\text{3D}}$ discussed in the previous section is depicted as the learning data in Fig. 1a. Second, the mode matrices and core tensor are obtained using the Tucker decomposition, as shown in Fig. 1b. Third, new components of the mode matrices corresponding to the predicted values of the multidimensional data are interpolated. For instance, to predict the multidimensional data for the new parameter value $p_{k'} \in {{\mathbb {R}}} \left( {p_k}< {p_{k'}} < {p_{k + 1}} \right)$, the interpolated value ${{\varvec{C}}}^{\text{predicted}}$ of the mode matrices are computed as follows:

Piecewise linear interpolation:

9 $$\begin{aligned} C_{k'v}^{{\text{predicted}}} = C_{kv}^{{\text{Tucker}}} + {M_{kv}}\left( {{p_{k'}} - {p_k}} \right) , \end{aligned}$$

Akima-spline interpolation^22^:

10 $$\begin{aligned} C_{k'v}^{{\text{predicted}}} &= C_{kv}^{{\text{Tucker}}} + {Z_{kv}}\left( {{p_{k'}} - {p_k}} \right) + \frac{{3{M_{kv}} - 2{Z_{kv}} - {Z_{k + 1,v}}}}{{{p_{k + 1}} - {p_k}}}{\left( {{p_{k'}} - {p_k}} \right) ^2} \\ & \quad + \frac{{{Z_{kv}} + {Z_{k + 1,v}} - 2{M_{kv}}}}{{{{\left( {{p_{k + 1}} - {p_k}} \right) }^2}}}{\left( {{p_{k'}} - {p_k}} \right) ^3}, \end{aligned}$$

where $k'$ is the index for the new parameter value, and $p_{k}$ is the parameter value of the *k*-th index in the parameter axis. Moreover, $M_{kv}$ is the slope of the segment from $\left( p_{k}, C_{kv}^{{\text{Tucker}}} \right)$ to $\left( p_{k+1}, C_{k+1,v}^{{\text{Tucker}}} \right)$, which is defined as

11 $$\begin{aligned} {M_{kv}} = \frac{{C_{k + 1,v}^{{\text{Tucker}}} - C_{kv}^{{\text{Tucker}}}}}{{{p_{k + 1}} - {p_k}}}\;\;\;\;\;\left( {k = 1, \ldots ,K - 1} \right) . \end{aligned}$$

To fit a cubic polynomial around the first and final parameters in the Akima-spline interpolation, the slopes preceding $p_1$ and beyond $p_K$ are extrapolated as follows:

12 $$\begin{aligned} {M_{ - 1,v}}= & {} 2{M_{0,v}} - {M_{1,v}}, \end{aligned}$$

13 $$\begin{aligned} {M_{0,v}}= & {} 2{M_{1,v}} - {M_{2,v}}, \end{aligned}$$

14 $$\begin{aligned} {M_{K,v}}= & {} 2{M_{K - 1,v}} - {M_{K - 2,v}}, \end{aligned}$$

15 $$\begin{aligned} {M_{K + 1,v}}= & {} 2{M_{K,v}} - {M_{K - 1,v}}. \end{aligned}$$

Furthermore, $Z_{kv}$ in Eq. (10) is defined as

16 $$\begin{aligned} {Z_{kv}} = \frac{{\left| {{M_{k + 1,v}} - {M_{kv}}} \right| {M_{k - 1,v}} + \left| {{M_{k - 1,v}} - {M_{k - 2,v}}} \right| {M_{kv}}}}{{\left| {{M_{k + 1,v}} - {M_{kv}}} \right| + \left| {{M_{k - 1,v}} - {M_{k - 2,v}}} \right| }}. \end{aligned}$$

Finally, the multidimensional data ${{\varvec{\mathcal {X}}}}^{\mathrm{{predicted}}}$ for the new parameter value $p_{k'}$ are predicted as

17 $$\begin{aligned} {{\mathcal {X}}}_{ijk'}^{{\text{predicted}}} = \sum \limits _{s = 1}^S {\sum \limits _{u = 1}^U {\sum \limits _{v = 1}^V {A_{is}^{{\text{Tucker}}}B_{ju}^{{\text{Tucker}}}C_{k'v}^{{\text{predicted}}}{G_{suv}}} } }. \end{aligned}$$

Although the prediction methods for the new parameter value are formulated based on three-dimensional data, the methods can be easily generalized to the prediction in the time or space axis and that of arbitrary *n*-dimensional data.

## Numerical example

In this section, the proposed method is applied to airbag deployment simulations for validation purposes. First, the analysis conditions of the airbag deployment simulations are summarized, and the construction procedure for learning the data from the results of the airbag deployment simulations is then presented. Second, ROMs are constructed based on the learning data to predict the airbag deployment behavior for new parameter sets. Moreover, the accuracy of the proposed method is investigated by comparing the prediction results with high-fidelity computational results. Finally, the response surfaces and Pareto frontier in multiple design spaces are efficiently predicted with the ROMs.

**Figure 2:**
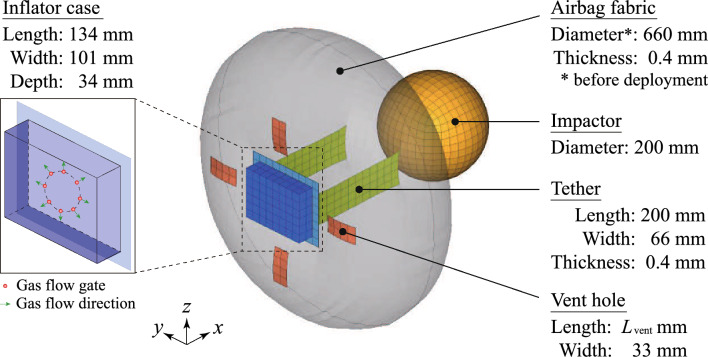
Airbag model for deployment and impact simulation. The airbag shape after inflation is displayed in translucent gray.

**Table 1:** Material parameters of the airbag fabric.

Material model		*MAT_FABRIC
Mass density ($\text{ton}/\text{mm}^3$)		$9.0 \times 10^{-10}$
Young’s modulus (MPa)	A-direction	300
	B-direction	200
	AB-direction	40
Poisson ratio (–)	BA-direction	0.2
Young’s modulus of liner (MPa)		60
Poisson ratio of liner (–)		0.35
Thickness ratio of liner (–)		0.1

### Model description for airbag deployment simulation

To construct learning data for ROMs, airbag deployment simulations were performed using the sample model^25^ produced by JSOL Corporation. The deployment and impact behavior of the airbag and impactor were computed using the finite element method, and the motion of the inflation gas was modeled by the corpuscular particle method^26^.

The finite element model is displayed in Fig. 2. The airbag fabric, tether, and vent holes were discretized by membrane elements, and the inflator case and impactor were discretized using shell elements. The model consists of 330 nodes and 3352 elements. The material parameters of each component are listed in Table 1. The fabric with the liner was set orthotropic elastic, and the inflator and impactor were considered rigid. The mass density of the inflator case was $8.0 \times 10^{-9}\,\text{ton}/\text{mm}^3$, and the weight *m* of the impactor was $5.0 \times 10^{-3}\,\text{ton}$. The elastic moduli and Poisson ratios of the inflator case and impactor were $2.0 \times 10^{5}\,\text{MPa}$ and 0.3, respectively. The contact of the fabric with itself and the impactor was considered. Furthermore, the inflator case was fixed, and the initial velocity in the *x*-direction of the impactor was $2.0 \times 10^{4}\,\text{mm}/\text{s}$.

Subsequently, analysis conditions for the corpuscular particle method are indicated. Air of 293 K and 1.0 atm was present inside and outside the airbag as the initial gas. The gates of the inflation gas were located concentrically at every $45^\circ$ on the *y*-*z* plane, as shown in Fig. 2, and the distance between the center of the inflator case and each gate was 30 mm. Nitrogen gas of 800 K flowed into the airbag from the gates for $T_{\text{inflation}}\,\text{s}$ at a mass flow rate of $2.0 \times 10^{-3}\,\text{ton}/\text{s}$. The properties of the air and nitrogen gas are displayed in Table 2. Their behavior was simulated using 20,000 particles (7000 for the initial gas inside the airbag and 13,000 for the inflation gas). This number of particles is high enough to describe a smooth pressure distribution on the fabric elements^27^. Moreover, the vent hole coefficient of Wang-Nefske leakage^28,29^ was 1.0.

**Figure 3:**
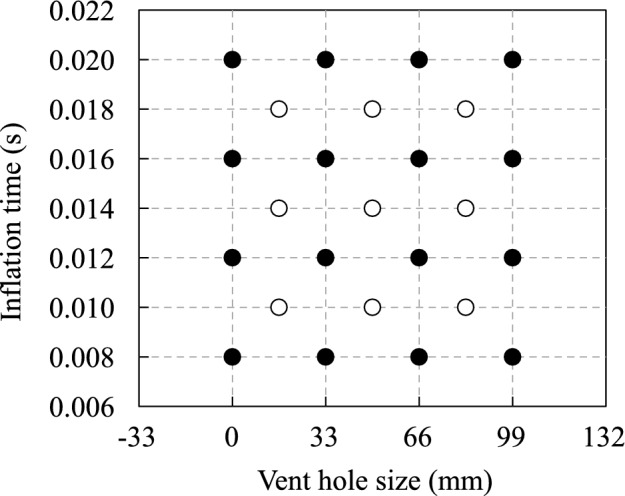
Parameter sets for learning and prediction data in the validation studies. Black and white circles indicate the learning and prediction data, respectively.

The length $L_{\text{vent}}$ of the vent holes and inflation time $T_{\text{inflation}}$ were considered as the design variables. The airbag deployment simulations with the parameters shown in Fig. 3 were performed to obtain the learning and validation data for prediction using our method. The termination time of the simulations was 0.1 s, and the time step size was $9.0 \times 10^{-7}\,\text{s}$. The simulations were performed with four cores of the Intel Xeon E5-2667v4 processors using massively parallel processing in the general-purpose finite element software LS-DYNA (R11.1.0).

**Figure 4:**
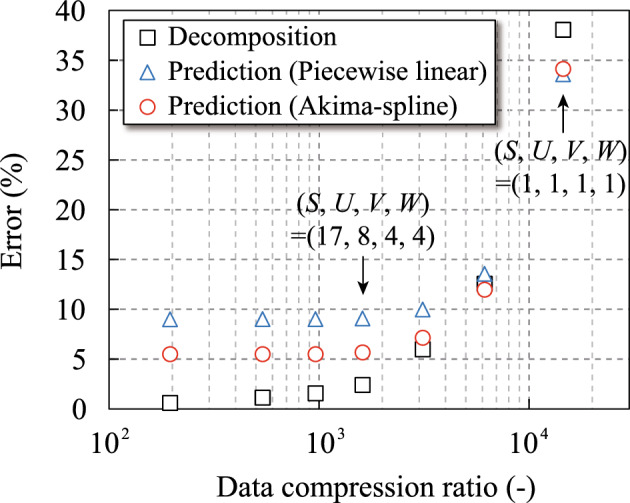
Relationship between the data compression ratio and the Tucker decomposition error.

**Table 2:** Properties of the initial and inflation gases.

		Air	Nitrogen
Molar mass (ton/mol)		$2.85 \times 10^{-5}$	$2.80 \times 10^{-5}$
Molar heat capacity parameter (–)	Constant	$2.88 \times 10^{4}$	$2.91 \times 10^{4}$
	Linear	− 0.894	− 2.46
	Quadratic	$9.29 \times 10^{-3}$	$1.04 \times 10^{-2}$

### Construction procedure for multidimensional learning data

We performed a numerical example to investigate the prediction accuracy of the deformation and energy absorption of the airbag, as well as the motion and maximum acceleration of the impactor. The time history response of each nodal displacement described the deformation behavior of the airbag motion of the impactor. Energy absorption was computed based on the initial and final velocities of the impactor. The maximum acceleration of the impactor was indicated from the time history response of its acceleration. Furthermore, the displacement and velocity of the impactor were computed by integrating its acceleration. Therefore, the multidimensional learning data ${\bar{{\varvec{\mathcal {X}}}}}^{\text{4D}}$ were constructed relying on the time history response of each nodal acceleration of the impactor and that of each nodal displacement of the airbag. The overline indicates that the variables are computed in this study using airbag deployment simulations. Note that the nodal acceleration of the impactor was employed because the velocity and acceleration computed by differentiating the displacement included noise and vibration. The learning data are four-dimensional data consisting of a time axis, space axis, first parameter axis, and second parameter axis representing the time steps, node numbers, vent hole size $L_{\text{vent}}$, and inflation time $T_\text{inflation}$, respectively. The matrix data ${{{\varvec{\bar{X}}}}_{kl}}^{\text{4D}}$ on the time and space axes included in ${\bar{{\varvec{\mathcal {X}}}}}^{\text{4D}}$ are displayed below:

18 $$\begin{aligned} {{{\varvec{{\bar{X}}}}}_{kl}}^{\text{4D}} = {\left[ {\begin{array}{*{20}{c}} {{{\varvec{d}}}_1^1}&{} \cdots &{}{{{\varvec{d}}}_1^n}&{} \cdots &{}{{{\varvec{d}}}_1^N}\\ \vdots &{}{}&{} \vdots &{}{}&{} \vdots \\ {{{\varvec{d}}}_i^1}&{} \cdots &{}{{{\varvec{d}}}_i^n}&{} \cdots &{}{{{\varvec{d}}}_i^N}\\ \vdots &{}{}&{} \vdots &{}{}&{} \vdots \\ {{{\varvec{d}}}_I^1}&{} \cdots &{}{{{\varvec{d}}}_I^n}&{} \cdots &{}{{{\varvec{d}}}_I^N} \end{array}} \right] _{kl}}, \end{aligned}$$

where *I* is the total number of time steps, *N* is the total number of nodes, and ${{\varvec{d}}}^n_i$ is the displacement vector of the *n*-th node at the *i*-th time step, which is defined as

19 $$\begin{aligned} {{\varvec{d}}}_i^n = \left[ {\begin{array}{*{20}{c}} {{d_x}}&{{d_y}}&{{d_z}} \end{array}} \right] _i^n, \end{aligned}$$

where ${{d_x}}, {{d_y}}$, and ${{d_z}}$ are the nodal displacements in the *x*, *y*, and *z* directions, respectively. Therefore, the total number *J* of the components of ${{\varvec{\bar{\mathcal {X}}}}}^{\text{4D}}$ on the space axis is three times larger than the total number of nodes, that is, $J = 3N$. Subsequently, the total numbers *K*, *L* of the components of ${\bar{{\varvec{\mathcal {X}}}}}^\text{4D}$ on the first and the second parameter axes are four, which is the number of levels of $L_{\text{vent}}$ and $T_{\text{inflation}}$ for the learning data, respectively. The matrix data ${{\varvec{X}}}_{k'l'}^{\text{predicted}}$ on the time and space axes included in ${{\varvec{{\mathcal {X}}}}}^{\text{predicted}}$ are described in the same way as Eq. (18). The total numbers $K', L'$ of the components of ${{\varvec{X}}}_{k'l'}^{\text{predicted}}$ on the first and second parameter axes are three when predicting for the white circles indicated in Fig. 3.

**Figure 5:**
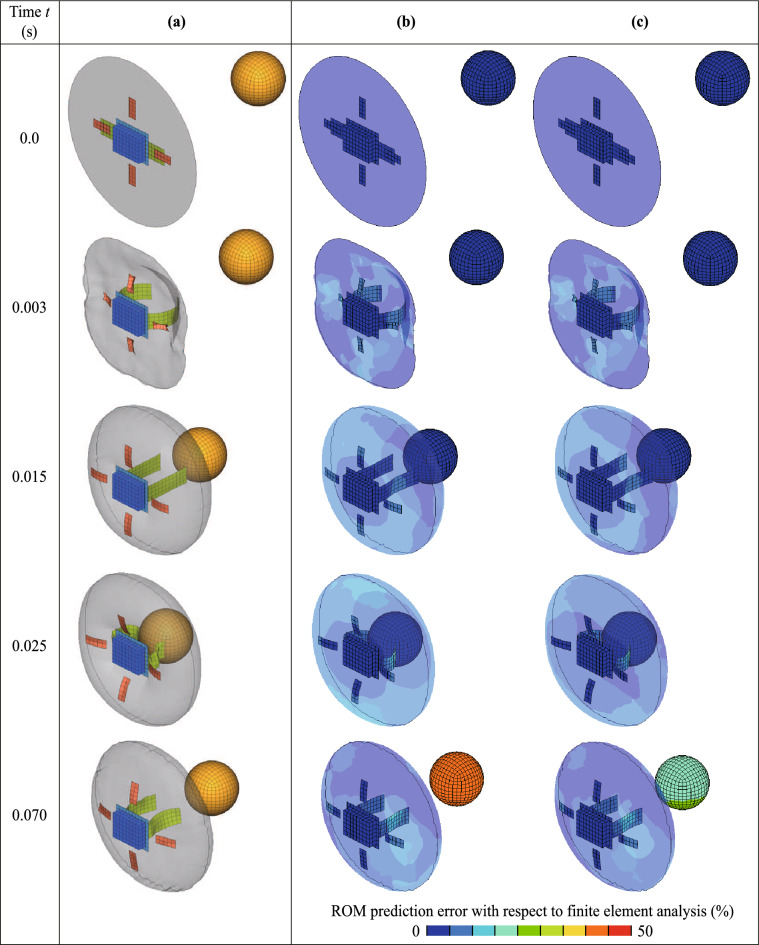
Comparison of airbag deployment behavior obtained by (**a**) finite element analysis and ROMs with (**b**) piecewise linear and (**c**) Akima-spline interpolation. Contour maps of errors regarding nodal displacements with respect to the finite element analysis are depicted in (**b**) and (**c**). Note that the behavior with the parameter set maximizing the prediction error using the Akima-spline approximation is displayed here.

**Figure 6:**
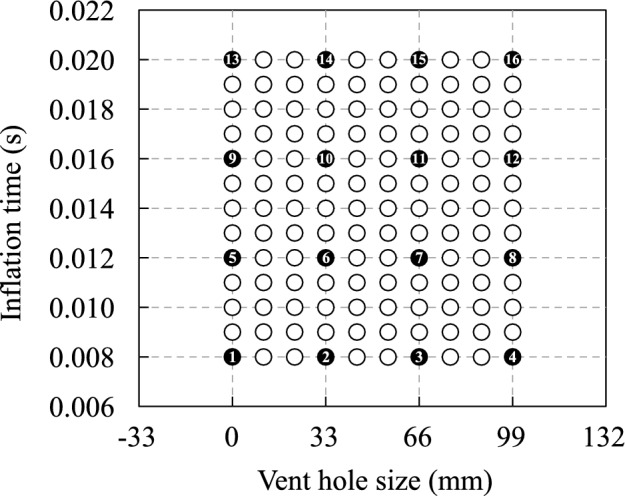
Parameter sets for learning and prediction data to predict the response surfaces and Pareto frontier. The black and white circles represent the learning and prediction data, respectively. The black circles are numbered to indicate the corresponding parameter sets of the learning data with their results.

**Table 3:** Computational results of the core consistency.

Size of core tensor				Core consistency (%)
*S*	*U*	*V*	*W*	
1	1	1	1	100
2	2	2	2	16.5
3	3	3	3	− 0.07

### Validation of prediction accuracy

By following the procedure explained in the previous section, the multidimensional learning data ${\bar{{\varvec{\mathcal {X}}}}}^{\text{4D}}$ were constructed from the results obtained by the airbag deployment simulation, as indicated in Fig. 3. Subsequently, the core consistency was computed with ${\bar{{\varvec{\mathcal {X}}}}}^{\text{4D}}$ using the N-way toolbox^30^ in the programming and numeric computing platform MATLAB (R2016b), as listed in Table 3. The results demonstrated that the Tucker model is suitable for this learning data because the core consistency becomes negative or close to zero when the size of the core tensor is larger than $\left( S, U, V, W \right) = \left( 2, 2, 2, 2 \right)$. In particular, the validation results with the Tucker model are shown below.

The relationship between data compression ratios and errors using Tucker decomposition with different sizes of the core tensor is summarized in Fig. 4. The sizes of the core tensor were determined using the function “hosvd” in the Tensor Toolbox^31^ with the following different tolerances: 0.01, 0.02, 0.03, 0.05, 0.1, and 0.2. The data compression ratio was computed by dividing the total number of components of the mode matrices and the core tensor by the total number of components of ${{\varvec{\bar{{\mathcal {X}}}}}}^{\text{4D}}$, which is defined as

20 $$\begin{aligned} {\mathrm{Data\,compression\,ratio}}= & {} \frac{{I \times J \times K \times L}}{{I \times S + J \times U + K \times V + L \times W + S \times U \times V \times W}}. \end{aligned}$$

Furthermore, the decomposition and prediction errors were obtained using the following equations:

21 $$\begin{aligned} {\mathrm{Decomposition\,error}}= & {} 100 \times {\left( {\frac{{\sum \nolimits _{i = 1}^I {\sum \nolimits _{j = 1}^J {\sum \nolimits _{k = 1}^K {\sum \nolimits _{l = 1}^L {{{\left( {{{\bar{{\mathcal {X}}}}_{ijkl}} - {{\mathcal {X}}}_{ijkl}^{{\text{Tucker}}}} \right) }^2}} } } } }}{{\sum \nolimits _{i = 1}^I {\sum \nolimits _{j = 1}^J {\sum \nolimits _{k = 1}^K {\sum \nolimits _{l = 1}^L {\bar{{\mathcal {X}}}_{ijkl}^2} } } } }}} \right) ^{\frac{1}{2}}}, \end{aligned}$$

22 $$\begin{aligned} {\mathrm{Prediction\,error}}= & {} 100 \times {\left( {\frac{{\sum \nolimits _{i' = 1}^{I'} {\sum \nolimits _{j' = 1}^{J'} {\sum \nolimits _{k' = 1}^{K'} {\sum \nolimits _{l' = 1}^{L'} {{{\left( {\bar{{\mathcal {X}}}_{i'j'k'l'}^{{\text{predicted}}} - \mathcal{X}_{i'j'k'l'}^{{\text{predicted}}}} \right) }^2}} } } } }}{{\sum \nolimits _{i' = 1}^{I'} {\sum \nolimits _{j' = 1}^{J'} {\sum \nolimits _{k' = 1}^{K'} {\sum \nolimits _{l' = 1}^{L'} {{{\left( {\bar{{\mathcal {X}}}_{i'j'k'l'}^{{\text{predicted}}}} \right) }^2}} } } } }}} \right) ^{\frac{1}{2}}}, \end{aligned}$$

where ${\bar{{\varvec{\mathcal {X}}}}}^{\mathrm{{predicted}}}$ is the airbag deployment simulation results using the parameter sets regarding the white circles indicated in Fig. 3. The data compression ratio was 14,545, which was the maximum when $\left( S, U, V, W \right) = \left( 1, 1, 1, 1 \right)$. As the size of the core tensor increased, the data compression ratio and errors decreased. However, the prediction errors reached their limits before reaching zero, whereas the decomposition error asymptotically approached zero. The optimal size of the core tensor was identical to $\left( 17, 8, 4, 4 \right)$ because the prediction errors did not change substantially when $\left( S, U, V, W \right) \le \left( 17, 8, 4, 4 \right)$. Additionally, the data compression ratio was 1614 when $\left( S, U, V, W \right) = \left( 17, 8, 4, 4 \right)$.

Tables 4 and 5 list the decomposition and prediction errors, respectively, regarding each parameter set for $\left( S, U, V, W \right) = \left( 17, 8, 4, 4 \right)$. Moreover, the decomposition and prediction errors were obtained by fixing the parameter indices $\left( k, l \right)$ in Eq. (21) and $\left( k', l' \right)$ in Eq. (22), respectively. The airbag deployment simulation results were predicted within 17% and 9% of the error using piecewise linear and Akima-spline interpolation, respectively. Furthermore, the errors tend to be large when the inflation time is small.

The deployment and impact behavior of the airbag and impactor when the prediction error using the Akima-spline approximation is at maximum; that is, at $L_{\text{vent}} = 82.5$ mm and $T_{\text{inflation}} = 0.01$ s, are summarized in Fig. 5. The errors in the contour maps show the difference in the nodal displacements between the finite element analysis and the proposed ROMs. The simulation results with the proposed ROMs agreed with those obtained by finite element analysis until $t = 0.025$ s. The errors of the impactor displacements at $t = 0.07$ s were large because of the irregular behavior of the impactor; that is, when the inflation time was short, the impactor hit the inflator. However, the error with the Akima-spline interpolation was approximately half of that with piecewise linear interpolation.

**Table 4:** Decomposition errors of the learning data using the Tucker model.

Vent hole size (mm)	Inflation time (s)	Decomposition error (%)
0	0.008	2.47
0	0.012	2.02
0	0.016	1.69
0	0.020	1.83
33	0.008	2.62
33	0.012	2.27
33	0.016	2.36
33	0.020	2.31
66	0.008	3.15
66	0.012	2.34
66	0.016	2.16
66	0.020	2.61
99	0.008	3.65
99	0.012	2.97
99	0.016	2.40
99	0.020	2.39

**Table 5:** Prediction errors of the airbag-deployment simulation results using the Tucker model.

Vent hole size (mm)	Inflation time (s)	Prediction error (%)	
		Piecewise linear	Akima-spline
16.5	0.010	9.37	4.02
16.5	0.014	3.32	4.62
16.5	0.018	4.28	5.45
49.5	0.010	16.46	8.73
49.5	0.014	6.77	5.37
49.5	0.018	7.09	4.68
82.5	0.010	15.94	8.78
82.5	0.014	3.58	2.92
82.5	0.018	3.96	3.31

### Prediction of response surfaces and Pareto frontier

**Figure 7:**
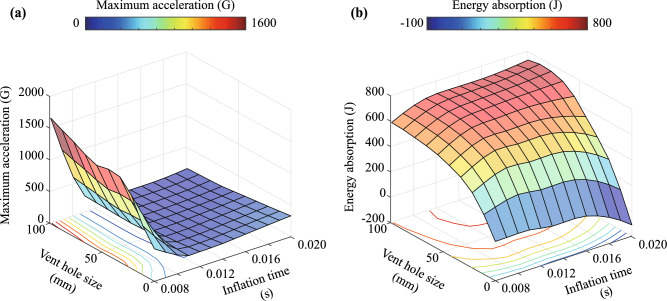
Response surfaces of (**a**) maximum acceleration and (**b**) energy absorption. They are predicted using reduced-order models based on the Tucker models and Akima-spline approximation.

The proposed ROMs can be employed in numerous parametric studies and response surfaces and Pareto frontiers in multiple design spaces. To predict the response surfaces and Pareto frontier, the deployment and impact behavior of the airbag and impactor were predicted using the Tucker models and Akima-spline approximation with the parameter sets indicated in Fig. 6. The maximum acceleration of the impactor and the energy absorption $E_\text{absorption}$ computed using the following equation for the parameter sets are depicted in Fig. 7. The values are calculated using the following equations:

23 $$\begin{aligned} E_{\text{absorption}} = \frac{1}{2}m\left( {v_{{\text{initial}}}^2 - v_{{\text{final}}}^2} \right) , \end{aligned}$$

where *m*, $v_{\mathrm{{initial}}}$, and $v_{\mathrm{{final}}}$ are the mass, initial velocity, and final velocity of the impactor, respectively. $v_{\mathrm{{final}}}$ was computed by integrating the acceleration of the impactor included in ${{\varvec{{\mathcal {X}}}}}^{\text{predicted}}$. The maximum acceleration of the impactor rapidly increased when the inflation time was shorter than approximately 0.01 s. The rapid increase in the maximum acceleration was caused by the collision of the impactor with the inflator when the inflation time was short. Figure 7a also shows that the maximum acceleration does not strongly depend on the vent hole size. Moreover, the energy absorption increased as the vent hole size and inflation time increased, as shown in Fig. 7b. The energy absorption was almost zero when $L_{\text{vent}} = 0$ mm, that is, without a vent hole. In addition, the energy absorption became negative when the inflation time was long without the vent hole because the gas inflation energy was transferred to the impactor.

**Figure 8:**
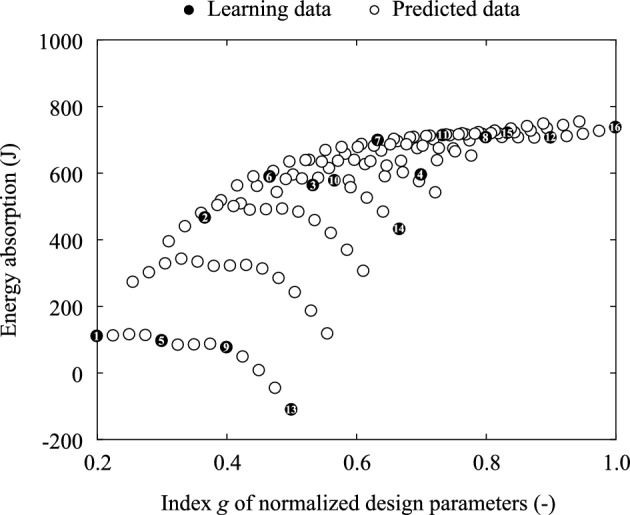
Relationship between the energy absorption and the design variables of the airbag. The black and white circles represent the learning and prediction data, respectively. The black circles are numbered to indicate the corresponding parameter sets of the learning data with their results.

The sensitivity of the vent hole size and the inflation time to the energy absorption was investigated by defining the following index *g*. Moreover, the relationship between the energy absorption and *g* is displayed in Fig. 8:

24 $$\begin{aligned} g = \frac{1}{2}\left( {\frac{{{L_{{\text{vent}}}}}}{{L_{{\text{vent}}}^{\max }}} + \frac{{{T_{{\text{inflation}}}}}}{{T_{{\text{inflation}}}^{\max }}}} \right) , \end{aligned}$$

where the maximum vent hole size $L_{{\text{vent}}}^{\max }$ is 99 mm, and the longest inflation time $T_{{\text{inflation}}}^{\max }$ is 0.02 s. *g* is the index that denotes the summation of the normalized $L_{{\text{vent}}}$ and $T_{{\text{inflation}}}$. The Pareto frontier regarding the energy absorption and product cost is depicted in Fig. 8 when the product cost of the airbag is proportional to the vent hole size and inflation time. Note that while the Pareto frontier with the learning data is unclear, the frontier with the predicted data using the ROMs is clear.

Finally, the computational costs are summarized. The average CPU time for the finite element analysis with a parameter set was approximately 114 min. Therefore, the total CPU time for the 130 parameter sets shown in Fig. 6 can be estimated to be  247 h. In contrast, the computational time with the ROMs was only approximately 7 min; that is, the response surfaces and Pareto frontier could be predicted 2000 times faster than the finite element analyses.

## Conclusion

In this study, a method for constructing ROMs for strongly non-linear problems involving contact and impact behaviors was proposed using tensor decomposition without tuning any parameters. The proposed method was verified and validated through airbag deployment simulations. Moreover, application to multi-objective optimization and suitable interpolation methods was investigated. First, finite element analyses of airbag deployment with 16 parameter sets were performed. Subsequently, four-dimensional learning data for the ROMs were constructed based on the time history response of each nodal acceleration of the impactor model and each nodal displacement of the airbag model. Second, the ROMs for the airbag deployment simulations were constructed by tensor decomposition of the learning data. Airbag deployment simulation results with nine other parameter sets were predicted using the ROMs with piecewise linear or Akima-spline interpolation. The error with the Akima-spline interpolation was approximately half of that with piecewise linear interpolation because the new parameter sets using the Akima-spline interpolation scheme could avoid unrealistic overshoots that occurred when using the standard cubic spline interpolation procedure. Finally, the response surfaces and Pareto frontier were efficiently predicted by computing the airbag deployment simulation results with 130 other parameter sets with the ROMs, which is 2000 times faster than full simulations required for finite element analyses. In conclusion, the proposed method can reduce product development cost and time by rapidly predicting the response surfaces and Pareto frontier. Our method is currently limited to inefficient grid sampling for gathering learning data; therefore, an extension of our method to random sampling will be considered in future work.

## Data Availability

The datasets generated during and/or analysed during the current study are available from the corresponding author on reasonable request.
